# Supplementary figures and images for: A Sporadic and Lethal Lassa Fever Case in Forest Guinea, 2019
Source: Viruses. 2020 Sep 23;12(10):1062. doi: 10.3390/v12101062 (PMC7598168; doi:10.3390/v12101062)

# Nucleoprotein, partial 630 nt

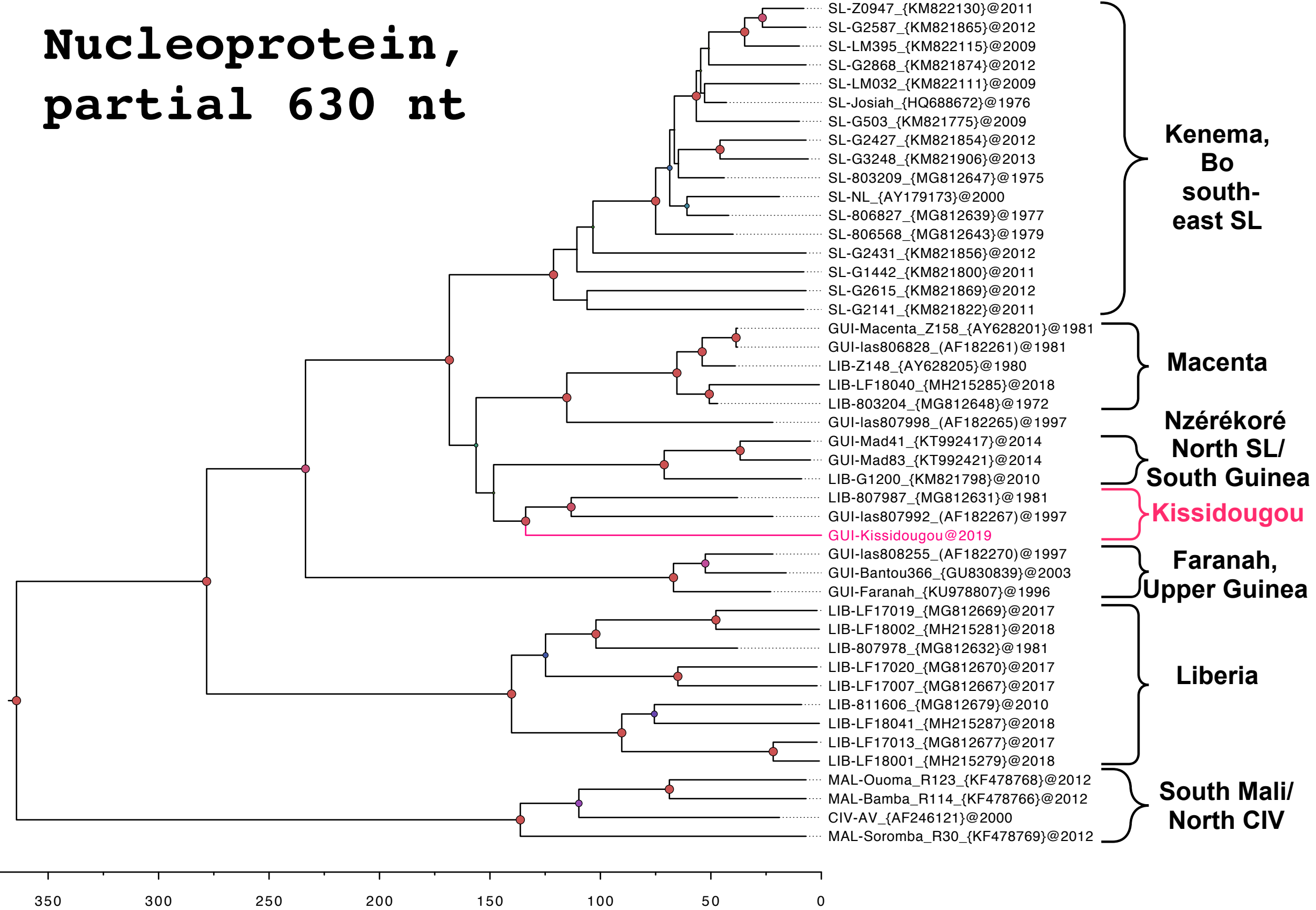

Supplement: Supplementary file 1 [file viruses-12-01062-s001.zip › viruses-926113-supplementary/figure S1 - copie.pdf]
